# Exploring the design and impact of integrated health and social care services for children and young people living in underserved populations: a systematic review

**DOI:** 10.1186/s12889-025-22508-7

**Published:** 2025-04-11

**Authors:** Chris Bird, Lorraine Harper, Syed Muslim, Derick Yates, Ian Litchfield

**Affiliations:** 1 Birmingham Women’s and Children’s NHS Foundation Trust, Birmingham, UK; 2 Nuffield Department of Primary Care Health Sciences, University of Oxford, Oxford, UK; 3 Department of Applied Health Sciences, University of Birmingham, Birmingham, UK; 4 Birmingham Health Partners, Birmingham, United Kingdom; 5 Queen Elizabeth Hospital Birmingham, University Hospitals Birmingham NHS Foundation Trust, Birmingham, United Kingdom; 6 Primary Health Care Corporation, Doha, Qatar

**Keywords:** Integrated care services, Children and young people, Health and social care, Health inequalities

## Abstract

**Objective:**

To explore the evidence for interventions that integrate child health and social care and support programmes and the impact they have on child health and wellbeing.

**Data sources:**

The Cochrane Library, Ovid Medline, Ovid Embase, Ovid Emcare, Ovid Health Management Information Consortium (HMIC) database, and Ovid Social Policy and Practice, Proquest Psychinfo and Ebscohost Cinahl.

**Eligibility:**

Peer-reviewed original research that described an intervention integrating health care and social support or care interventions for children and young people (CYP) up to the age of 18 years in high-income countries. All databases were searched from inception to August 2023.

**Data extraction and synthesis:**

16 studies were identified: 9 quantitative studies including 4 RCTs, 5 qualitative studies and 2 mixed methods studies. Studies were assessed for quality and a narrative review performed. Study heterogeneity meant a meta-analysis could not be completed.

**Results:**

For the purposes of clarity and understanding we collated the identified studies bv mode of delivery. In doing so we determined three main models of delivering integrated health and social care services: *Targeted support for vulnerable groups*, where the provision of packages of interventions focussed on target populations, this showed potential for decreasing the need for social support in the long-term but with limited evidence for reducing referrals into other services. These types of service were more successful in meeting specific objectives such as lower rates of smoking, and reducing repeat pregnancies; *Collaborative health and social support*, which typically collocated health and social care practitioners, demonstrated improved collaborative working but with little impact on workload, job satisfaction, or service delivery; and *School centred health and social care*, which were based in educational facilities and improved some aspects of CYP wellbeing and physical health but with concerns they added to teacher workload.

**Conclusions:**

Integrated health and social support programmes offer promising solutions to addressing health inequity in children and young people in underserved populations. However, more robust and consistent study designs are needed to guide researchers and policy makers in their implementation and evaluation.

**PROSPERO registration:**

CRD42023399907

**Supplementary Information:**

The online version contains supplementary material available at 10.1186/s12889-025-22508-7.

## Introduction

Children, young people (CYP) and their families living in high income countries face mounting challenges to their health and well-being, as the prevalence of chronic conditions, obesity, and mental ill health continues to increase [1]. These challenges are exacerbated in underserved populations i.e., minoritized and economically-deprived communities [2, 3], by a range of socio-economic and cultural pressures that inhibit access and utilisation of primary or preventative health care services [4–7]. This has led to a widespread rise in children’s attendances to emergency departments frequently due to conditions that could be more effectively treated in community settings [8–12].

Previously combinations of health and social care have been accessed via child support services and not through the healthcare system and as such opportunities to provide broader support can be lost [13, 14]. The need for more responsive, culturally sensitive primary care for CYP from underserved populations has led to efforts in North America, Europe and Australia to prioritise more localized service delivery that integrates several strands of health and social care and places a greater emphasis on public and preventative health [13, 15–20]. The integrated services that have emerged are delivered by various combinations of health care providers, social care practitioners, community advocates, and public institutions, and situated in a range of central and localised clinical and locality-based settings [21, 22]. Together they share the aim of providing widely accessible health and social care for CYP and their families that can help treat and manage acute and chronic health care alongside the necessary social support that can help mitigate the social determinants of ill-health such as poor housing, domestic violence, or food poverty [22, 23].

However, despite widespread investment in these systems in countries such as the United Kingdom [24], evidence of the benefits of integrating health and social care remains inconsistent, particularly amongst underserved CYP [25, 26]: Little is known of which integrated models are most effective, including the precise combination of services, the specific outcomes they improve, or the impact on the surrounding health economy [27, 28]. To the best of our knowledge this systematic review is the first that has collated and examined the impact of these integrated health and social care services on CYP in underserved populations. It suggests a typography of the various service models employed and presents the qualitative and quantitative evidence of the effectiveness of each.

## Methods

### Study design

This work consists of a systematic review of qualitative, quantitative studies and mixed methods studies [29]. We used the PerSPEcTiF model to frame the review question (see Table 1) [30] and followed the Preferred Reporting Items for Systematic Reviews and Meta-Analyses (PRISMA) guideline [31]. The study is registered on PROSPERO (International Prospective Register of Systematic Reviews: CRD42023399907) [32].

### Search strategy

The review question was designed using the PerSPEcTiF question framework, to enable the search to best identify a set of relevant abstracts of interest, and the database search structure followed a Population, Exposure, Outcomes (PEO) approach (see Supplementary File 1). The following databases were searched: Cochrane Library, Ovid Medline, Ovid Embase, Ovid Emcare, Ovid Health Management Information Consortium (HMIC) database, Ovid Social Policy and Practice, Proquest Psychinfo and Ebscohost Cinahl.

**Table 1:** Review framework using perspectif (*Booth et al.*,* 2019*)

Full Review Question (using PerSPEcTiF framework)	
Do interventions that integrate health and social support impact the health and wellbeing of children and young people (CYP) from underserved areas?	
**Per**spective	From the perspectives of both families and CYP from underserved areas who use the service, those who deliver the service and outside observers.
**S**etting	Interventions where healthcare and social support programmes for CYP in underserved areas are described/presented as ‘integrated’, including qualitative studies, quantitative studies (RCTs, cohort, observational, quasi experimental), no date limit (exclude case reports, reviews, commentary).
**P**henomenon of interest/problem	Impacts on a wide range of outcomes on health (preventive, acute, chronic health issues) and wellbeing (e.g. anxiety) for CYP.
**E**nvironment	High income countries with a particular focus on interventions in underserved areas, with a focus on underserved populations similar to those in the UK (e.g. Europe, New Zealand, Australia).
**C**omparison	Standard care, if a comparator available.
**Ti**me	CYP < 18 years, at any point during their childhood (e.g. infant, pre-school, primary and secondary school age).
**F**indings	Impacts on child health and wellbeing, e.g. school attendance, asthma control, including: qualitative – patient/professional value/experience of service; addressing social determinants of health; quantitative – cost effectiveness, primary and secondary care use, school attendance, social determinants of health. Given the complex nature of these interventions, outcome measures likely to be heterogeneous.

### Inclusion criteria

Studies were eligible for inclusion if their focus was health and social care (including the provision of social work, personal care, protection or social support [33]) delivered as an integrated service distinguished by its coordinated, planning, commissioning and provision [34], and targeted “underserved populations” defined as those groups possessing “health differences that are avoidable, unnecessary, and unjust” [35]. All databases were searched from 1946 to 31st August 2023 with no limits in relation to study, publication type, language or date of publication. The search identified a combination of relevant subject headings within those databases using a controlled vocabulary; MeSH in Cochrane, Medline and Cinahl. Emtree in Embase and Emcare and APA Thesaurus of Psychological Index Terms in PsychInfo combined with keywords and free text word variations. Proximity operators were used to maximise the efficiency of the search strategy when searching for phrase variations. The full search strategy is available in Supplementary File 1.

### Study selection and assessment of quality and bias

Identified studies were collated and managed using Endnote and Covidence software [36, 37]. Two independent reviewers (CB and SM) identified relevant papers by reading titles and abstracts and disagreements were resolved through joint review and consensus. Full texts for these papers were retrieved when there was insufficient information in the abstract to form a judgement. One reviewer extracted data from the selected papers using a data extraction form (CB), which was then checked by a second reviewer (SM). The data extracted included author, country, aims, sample size, study design and results [38].

Both reviewers assessed study quality and risk of bias and scored each study using as appropriate:

- the Critical Appraisal Skills Programme qualitative studies, a checklist which takes a structured approach to ensure “methodological rigour, validity, and relevance” [39].
- the Effective Public Healthcare Panacea Project’s quality assessment tool for quantitative studies, which scores selection bias, study design, risk of confounding, blinding, data collection, drop-outs, integrity of the study, with a global rating of either “strong”, “moderate” or “weak” evidence [40].
- McGill University’s Mixed Methods Appraisal Tool for mixed methods studies, guiding reviewers to rate five different study designs in any mixed methods study, which include: qualitative research; randomised controlled trials; non-randomised studies; quantitative descriptive studies; mixed methods studies [41].

### Data analysis

Data were collated, organised, and analysed according to the shared characteristics of the service they delivered. If the data were available a meta-analysis of patient outcomes would have been conducted, in its absence a narrative synthesis was conducted using qualitative data augmented with quantitative data where available [30, 41]. The narrative synthesis followed best practice, exploring relationships in the data within and between studies, and iteratively refining its interpretation to arrive at the structured description of the findings within each of the three models of integrated health and social care identified [42]:

## Results

### Study characteristics

**Fig. 1:**
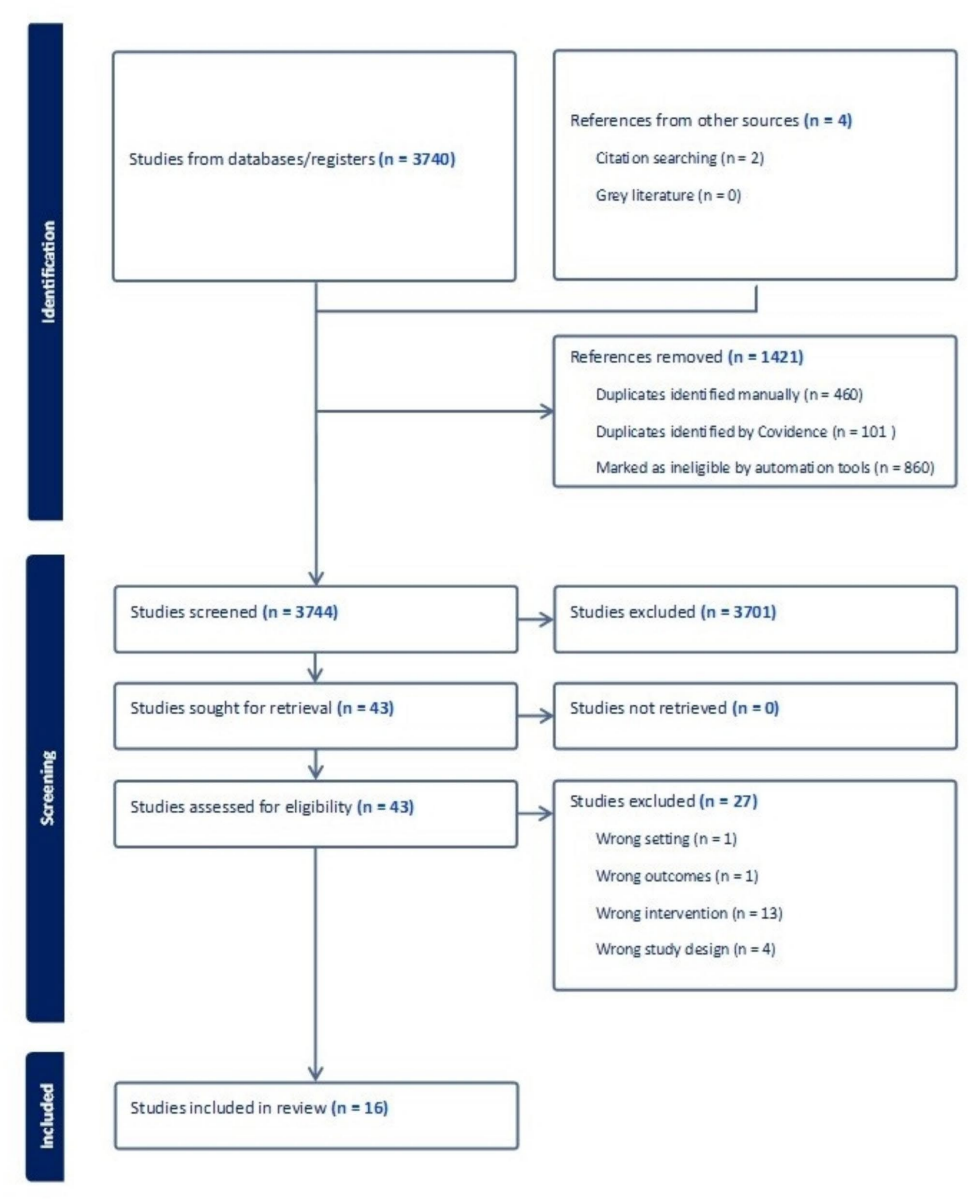
PRISMA flow diagram

A total of 3,741 studies were imported for screening and four studies were found via hand searches. 1,421 duplicates were removed, 3,701 studies were screened, 43 full text studies were assessed for eligibility and 16 studies were included in the review. Studies were excluded because they were either of an incorrect intervention (*n* = 13), study design (*n* = 4), setting (*n* = 1), outcome (*n* = 1), population (*n* = 1), or reported no results of impact (*n* = 7). These are described in the PRISMA flow diagram (see Fig. 1) [31]. Five qualitative, nine quantitative (including four randomised controlled trials (RCTS), two mixed methods were included. All studies were carried out in Australia, North America, or Western Europe. The key characteristics and main findings of each study are further described in Table 2.

**Table 2:** Key characteristics and main findings of included studies

Author,	Country	Title/ aims	Sample size/ characteristics	Study design Quality assessment (assessment tool)*	Outcomes	Results**	Type of integrated service model
Barnett et al., 2020, [43]	USA	The impact of 7 “wellness navigators” on families experiencing adverse childhood experiences.	99 mainly Latinx carer-infant family dyads participated (126 eligible)	Retrospective, mixed methods – quantitative and qualitative Weak (MMAT)	Quantitative – number and type of referrals to support services made for each family. Qualitative – providers’ and caregivers’ experience of the intervention	Quantitative – wellness navigators made referrals for 53% of families, with a mean of 5.52 referrals per family (SD = 7.93). Referrals mainly for health insurance, childcare and housing. Qualitative – increased access to services, better holistic care	Targeted support for CYP and their families
Rinehart et al., 2021, [44]	USA	Use of a screening tool to help paediatricians identify CYP attending clinics needing social support.	300 families who attended a paediatric clinic and answered screening questionnaire	Retrospective, observational study Weak (EPHPP)	Primary outcome number of referrals made, Secondary outcome The Description of social needs identified, number of referrals made	Referrals were accepted by 80.3% of families who screened positive for need. Of 300 families 58.7% had at least one unmet need (average 1.4 per family). Commonest issues were home environment (40%), tobacco exposure (29.3%) and food insecurity (20.6%).	Targeted support for CYP and their families
Browne et al., 2001, [45]	Canada	Provision of additional support for families on income support	765 families enrolled, 53% of families had dropped out by year 2 of the 4-year study	Five-arm, randomised controlled trial, comprehensive care (health promotion, employment training and parenting skills) or one of the three interventions compared to self-directed (standard) care. Moderate (EPHPP)	Primary outcome Impact on parental mood disorders, Secondary outcome child behaviour disorders, child competence, social independence, use of health and social services	No difference between arms for any outcomes except 15% more families who had comprehensive care no longer needed social assistance after 12 months compared to families in the self-directed (standard) arm 53% of families had dropped out by year 2 of the 4-year study	Targeted support for CYP and their families
Drummond et al., 2016, [46]	Canada	Exploring three different service delivery models linking low-income families with health and social support.	1,168 families receiving state assistance randomized to the 3 different interventions and standard care, 3-year follow-up	Randomized, two-factor, single-blind, longitudinal effectiveness trial Families were randomised to (1) Family Healthy Lifestyle and Family Recreation (comprehensive) (2) Family Healthy Lifestyle (3) Family Recreation and (4) Standard care. Strong (EPHPP)	Primary outcome – number of family linkages to health and social services. Secondary outcome -s – family experience, cost, family health and functioning	Significant difference for child development linkage in Family Healthy Lifestyle alone (RR 3.27, 1.59–6.74) and for health care linkage in comprehensive package (RR 1.27 (1.06–1.51)	Targeted support for CYP and their families
Cox et al., 2012, USA [47]	USA	Creating a medical home model for adolescent mothers and their children	181 adolescent mothers followed up at 12 and 24 months	Prospective, single cohort study exploring preventive care, pregnancy and psychosocial support through outreach services at adjacent hospital. Moderate (EPHPP)	Primary outcome – number of mother and child health visits Secondary outcomes (preventative care) contraception use, repeat pregnancies, child immunizations; (life skills) mother in school/ graduated, employment, receiving state aid, paternal support	At final follow-up lower repeat pregnancy rate but lower contraceptive use and no difference on immunization rate Lower percentage of mothers living with their own parents, higher paternal financial support,	Targeted support for CYP and their families
Garg et al., 2015, [48]	USA	Use of ‘WE CARE’ screening tool for mother-infant dyads	366 families from deprived area of Boston, 42 in each cluster (4 intervention, 4 standard care) with infants followed up to approximately 12 months	Cluster randomised controlled trial which screened for six basic needs (child care, food security, household heat, housing, parent education, employment) and initiated referrals to community resources for unmet needs Weak (EPHPP)	Primary outcome - number of referrals to a community resource made for infants by age of 12 months	68% of families in both arms had =/> 2 unmet basic needs. More mothers in WE CARE arm received a referral compared to standard care (70% vs. 8%, OR 29.6, 14.7–59.6) while more mothers in WE CARE arm enrolled in a community resource (39% vs. 24%, OR 2.1, 1.2–3.7).	Targeted support for CYP and their families
Jones et al., 2020, [50]	UK	Health and social support (midwives, family facilitators, nursery nurses, speech and language therapists) for young parents	Parents (aged 16–24 from 17 weeks of pregnancy) 568 families over 2 years	Retrospective cohort study Weak (EPHPP)	Outcomes Smoking, alcohol and diet during pregnancy, breastfeeding, screening for adverse childhood events, number and outcome of referrals to social services	68.2% families completed JIGSO programme; median midwife visit 6 antenatal and 3 postnatal; 25.5%v clients stopped smoking during pregnancy (6% standard care), no improvement in breastfeeding rates, improved confidence in parenting, significant association between children discharged from social services and number of JIGSO visits	Targeted support for CYP and their families
Garg et al., 2023, [49]	USA	Using a patient navigator with a screening tool for seven basic needs (child care, education, employment, food security, household heat, housing, language)	878 parent-child dyads who presented for a newborn assessment at a participating community healthcare centre	Type 1 hybrid effectiveness cluster randomised controlled trial Weak (EPHPP)	Primary outcome Evaluated number of families referred by to patient navigators (and where to) services referred for; Secondary outcomes impact on adherence to well child visits and immunisation uptake; impact on ED attendances and hospitalisations	Only 28.9% of families were screened for needs, of whom 20% were referred to a patient navigator – one of the three intervention clusters was excluded due to contamination. There was no significant difference for adherence to well child visits and ED visits and hospitalisations were significantly higher in the intervention arm	Targeted support for CYP and their families
Purcal et al., 2011, [51]	Australia	The impact of direct funding for partnerships on integration of early years programmes (0–5 years) combining health and social support	Staff surveyed at 41 “early years” centres at 2 time points and follow-up interviews (10 centres, 222 interviews	Retrospective, mixed methods – quantitative and qualitative Weak (MMAT)	Experience of partnership, partnership activities	Initial survey response rate 20%, second wave response rate 81% - integrated working perceived to improve. Integration significantly increased interagency referrals and training	Collaborative health and social support
Martinussen et al., 2017, [52]	Norway	Evaluated social and healthcare workers’ experience of integrating health and social care for CYP following reorganisation of “early years” services.	Questionnaires delivered to all employees delivering the service (response rate 83%)	Survey Weak (EPHPP)	Experience on job demands and resources, collaboration, burn out and job satisfaction	Decreased conflict and improved collaboration but no change in workload or job satisfaction	Collaborative health and social support
Saxe-Custack et al., 2018, [53]	USA	Explored caregiver experiences of co-locating a paediatric clinic in a farmer’s market with a healthy food prescription for CYP.	32 caregivers attending a paediatric clinic	Qualitative study Strong (CASP)	Caregivers’ experience of the programme	Families valued location close to home, the food prescriptions aided food security and prompted healthier eating habits, but some parents perceived the prescription as lacking choice	Collaborative health and social support
Murillo et al., 2022, [54]	USA	How a lawyer co-located in a paediatric clinic affected paediatric practice	20 paediatricians, 20 parents/ guardians	Qualitative study Moderate (CASP)	Experience of paediatricians and families working with co-located lawyer	Greater awareness and understanding of social determinants of health and health-harming legal needs	Collaborative health and social support
Elsenburg et al., 2022, [55]	Netherlands	How funding to integrate health and social support changed quality of life and psychosocial problems among CYP at schools in a deprived area of Amsterdam	614 CYP aged 7–13 years from 5 schools over 2 years	CYP surveyed at time intervals (longitudinal) Weak (EPHPP)	CYP quality of life measured using KIDSCREEN-10 questionnaire	Health related quality of life appeared to improve but no difference for physical or psychosocial wellbeing. Scores went down after funding ended.	School centred health and social care
Elsenburg et al., 2023, [56]	Netherlands	How funding to integrate health and social support changed quality of life and psychosocial problems among CYP at schools in a deprived area of Amsterdam	15 school principals	Qualitative study Strong (CASP)	Which initiatives chosen by schools, what impact these had and how they differed.	Indications of improved teaching climate, health and socioemotional health of students;; reported negative impact on school workload, coordination of care and parent involvement in education.	School centred health and social care
Sanford et al., 2020, [57]	Australia	The role of nurses in improving access to health care, health promotion, and local support.	14 participants from 9 schools (seven primary and two secondary schools)	Qualitative study Teacher focus groups (x4) nursing focus group (x1) Strong (CASP)	Experience of teachers and nurses of the intervention	Nurses provided useful bridge between services, better sharing of information, identification of unmet needs (e.g. mental health)	School centred health and social care
Sanford et al., 2022, [58]	Australia	Explored nurse and learning support staff experiences in implementing an integrated school-nursing model.	25 participants from 6 schools	Qualitative study Support Team focus groups (x4) Nursing focus group (*n* = 5) Strong (CASP)	Experience of Learning Support Team workers and nurses of the intervention	Challenges reported in defining role of nurse, recognised importance of involving all stakeholders early	School centred health and social care

*CASP = Critical Appraisal Skills Programme checklist for qualitative studies; EPHPP = Effective Public Healthcare Panacea Project’s quality assessment tool for quantitative studies; MMAT = Mixed Methods Appraisal Tool

** RR = relative risk; OR = odds ratio

### Integrated health and social care models

We identified three types of integrated health and social care services: *Targeted support for CYP and their families*, where specified groups with additional needs were identified and then receive a range of health and care interventions [43–50]; *Collaborative health and social support*, involving health and social care organisations work together to deliver shared and complementary services [51–54]; and *School centred health and social care*, in which health and social care services embedded or directly linked to educational facilities [55–58]. A summary of the key characteristics and main findings of each of the types of integrated care model can be found in Table 3 and below we summarise the results of the studies identified within each.

**Table 3:** Summary of the models of integrated health and social care designed to support underserved CYP

Integrated model	Definition	Location	Key characteristics	Content of intervention(s)
*Targeted support for CYP and families*	A service based on identification of a particular target group or population and then offered one, or a combination of several interventions consisting of health and social care intended to impact a pre-specified outcome(s).	Clinical, community, or domestic settings	Identification of those with direct responsibility for CYP health i.e., individual CYP, their carers and/or families. Single, or a package of, interventions delivered by multiple care organisations reflective of clinical and social need of target groups	Interventions include education (numeracy and literacy), employment training, parenting skills, and health promotion and preventative care (e.g., healthy eating, smoking cessation, vaccination, contraception)
*Collaborative health and social support*	An integrated service provided by health and social care organisations and their practitioners offered to localised populations	Predominantly collocated in a shared (community) health care facility	The sharing of key aims, infrastructure, and financial responsibilities between health and social care organisations	A wide range of (preventative) health and social care and support. Including clinical care, legal counselling, health promotion and preventative care, oral hygiene, and mental health services
*School centred health and social care*	Health and social care services embedded or otherwise linked with the delivery of primary and secondary education to populations that include CYP from underserved groups	Predominantly delivered within primary and secondary school premises	Health and social care practitioners and/or public health initiatives embedded within or linked to local schools	Programmes that connect CYP and their parents with social and culturally sensitive health care and social support. Including health promotion and preventative care, and signposting to social support services

### Targeted support for CYP and their families

Target groups consisted of CYP and their families [44–46], or (young) mothers with infants [43, 47–50]. They were identified via bespoke screening tools [48, 49], through their existing or previous use of social care or support [43], and an actual or proxy measure of low-income or deprivation [44–46]. The interventions were typically delivered in community based care centres or clinics [43, 44, 48–50], or in two instances the CYP’s home [47, 50]. Four studies targeted CYP and their families, three were recognised as requiring additional needs by direct or proxy measure of deprivation [44–46] and one by previous contact with social care services [43].

At a single clinic in a deprived district in East Harlem just under 60% of participants had at least one unmet need relating to housing, tobacco exposure, or food insecurity with 80% successfully referred to the appropriate social support as a result [44]. A multi-arm randomised controlled trial (RCT) set in Canada identified vulnerable families by a locality-based deprivation score that accessed a range of interventions including various combinations of health promotion, parenting skills, and employment training, with the published interim analysis indicating that those receiving the intervention were less likely to need social assistance 12 months later [45]. A second RCT, also in Canada, recruited participants from locality-based deprivation scores described the impact of a range of family-based lifestyle and recreational interventions with significant improvements in engagement with child development services (RR 3.27, 1.59–6.74) and health care (RR 1.27 (1.06–1.51) [46]. However, over half of families receiving the intervention dropped out after two years and the authors observed that integrating the work of the existing agencies did not address longstanding shortages in service capacity [46]. Barnett et al. reported that Latinx carer-infant dyads identified by previous contact with social support subsequently had increased referrals to organisations providing health insurance, childcare and housing [43].

Two studies targeted young mothers [47, 50]: One, set in the USA, that integrated support from hospital staff and social workers into a “medical home” model and reported they were less likely to live with their own parents, have a repeat pregnancy and received greater paternal financial support [47]. The other study set in Wales (UK) consisted of health and social support from a team of midwives, family facilitators, nursery nurses, and speech and language therapists [50]. They reported reduced smoking rates, and improved confidence in parenting though no increase in breastfeeding [50].

Two related studies targeted mothers (of any age) and their infants with unmet needs [48, 49]. They found that those identified using the tool were significantly more likely to receive a referral to community (social) services, though only half of those actually received additional support [48]. Adapting the screening tool to incorporate multiple languages and linking participants with a patient-peer navigator increased the likelihood of an ED visit or hospitalisation [49].

### Collaborative health and social support

The facilitated collaborations consisted of community-based co-located social support and health care services set in Australia [51], USA [53, 54], and in Norway an organisational-level collaborative service [52]. Purcal et al.’s Australian study of state funding for integrating professional health and social support found a significant increase in inter-agency referrals but no impact on planning, service delivery or co-location, according to senior managers, managers and frontline staff [51].

Two single-centred, US-based studies described social support interventions co-located in paediatric clinics: one provided a fresh food prescription though families felt that they aided food security but also felt the options were constrictive and would have preferred vouchers [53]. In the second a paediatric clinic provided a lawyer to tackle health-harming legal needs such as those relating to housing, utilities, guardianship, education and benefits [54]. The qualitative data indicated greater confidence and trust, from CYP and families, in clinical staff, who in turn reported improved awareness and understanding of the social determinants of their patients’ health [54]. Martinussen et al.’s survey of Norwegian health professionals following the re-organisation of services to better integrate social care found improved collaboration but did little to improve job satisfaction or reduce workload [52].

### School centred health and social care

Of the four studies identified, two evaluated a single intervention in Australia that comprised of linked nurses within primary and secondary schools situated within economically disadvantaged locations in Australia evaluating [57, 58]; and two in the Netherlands exploring the impact of central funding on a range of small scale health and social care packages determined by primary school leaders [55, 56].

The Australian qualitative studies explored the views of nursing link workers and teachers and learning support workers working together in primary and secondary schools in Australia [57, 58]. Both school and nursing staff reported that care navigation improved, with better information sharing and identification of unmet needs (e.g. mental health) but there were challenges in defining the nurses’ roles and how they worked alongside school support staff [57, 58].

The two studies that evaluated the 2-year programme in the Netherlands, where government provided €125,000 to schools in economically disadvantaged areas to fund their choice of interventions with the premise they would integrate health and social support for CYP aged 7–13 years [55, 56]. The first described the results of a longitudinal survey of pupils which indicated that health related quality of life and psychosocial problems improved, and though the scores used to measure wellbeing displayed little variation over time, they did drop off once funding ended [55]. The second study of stakeholder perceptions, described perceptions of improved wellbeing, physical health and classroom behaviour, though the school leaders were concerned about sustained impact due to the impact of interventions on teacher workload, and coordination of care [56].

## Discussion

### General findings

This review provides valuable and novel insight into the various attempts at integrating health and social care for the benefit of CYP and families from underserved populations. Of the papers identified, we were able to produce a typology that categorised the studies into three different models of service delivery. *Targeted support for CYP and their families*: Which involved the initial identification of vulnerable groups and the subsequent provision of various combinations of interventions targeted at their specific needs. This showed potential for decreasing the need for social support in the long-term, but with limited evidence for improving intervention specific outcomes such as referrals into other services, lower rates of smoking, reduced repeat pregnancies, or discharge from social services. *Collaborative health and social care* consisting of community collocated health and social care and support services. These demonstrated some improvement in collaboration between previously disparate services including co-locating social support in paediatric clinics, and the introduction of legal practitioners into the team, though there was no impact on workload, job satisfaction, or service delivery. *School centred health and social care* consisting of linking school environments with health and social support interventions improved some aspects of CYP wellbeing and physical health, but senior educational staff reported increased teacher workload.

### Specific findings

#### Targeted support for CYP and families

Target groups were readily identified but the evidence of the various approaches used to identify these groups was inconsistent, with some improvements reported in streamlining referrals into other services [44, 45, 48, 49], reducing the number of repeat pregnancies [50], or smoking [53]. Similar approaches targeting deprived families (though without integrating health and social care), have also shown promise in promoting healthy behaviours [21, 59] for example raising awareness of oral health in the UK [60], reducing childhood obesity [59, 61].

The lack of definitive evidence of the benefits of health and social care interventions for underserved CYP can be attributed to well-known socio-cultural barriers that prevent their accessing health and social care even when specifically targeted at these populations [4–7]. It appears time to recognise the value of using alternative means of improving outreach to the most vulnerable such as via housing associations [62, 63] or homelessness charities [64]. These novel routes of engagement can then result in meaningful co-design of health and social care interventions, supporting an increase in community ownership of the intervention and reducing the stigma of those that subsequently access the support [65]. Future interventions might also be better supported by embedding peers or community connectors in the delivery or facilitation of the service, to help address the persistent issues of mistrust and engagement with mainstream health and social support services [66, 67]. There is also the difficulty in evidencing the success of such interventions due to the broader difficulties in researching these groups hindered by frequent changes of address; concern over misuse of data, and language and cultural barriers [68].

#### Collaborative health and social support

The studies we uncovered reported limited benefits of collaborative health and social care including more effective referral into social support services and increased job satisfaction [51–54]. The majority of previous work that has explored inter agency collaborative working has focused on creating teams of primary and secondary care clinicians [69, 70]. What remains less well explored and understood is how to combine health and social care services, and despite the promise of benefits for patients and staff organisational barriers persist particularly with social care being less though early indications suggest there is a lack of understanding and recognition of the role of social care [25, 26].

Professional partnership working between different disciplines requires bridging differences in training, aims, and work practices of health and social care practitioners [71]. Colocation of services can help and supports better communication, understanding, and mutual learning [72, 73]. However, if the integration of health and social care services is to be sustained in the long-term then fundamental issues around professional identities and boundaries need to be addressed [74]. This requires changes in training and education to better ensure such partnership working remains safe and effective [75] with techniques such as ‘inter-disciplinary observation’ recognised as an aid to fostering mutual respect, greater job satisfaction and workforce retention [76–79].

#### School centred health and social care

Delivery of health and wellbeing through schools has been promoted globally for several decades and was recognised as part of the World Health Organisation’s 1986 Charter on Health Promotion which asks that schools constantly strengthen their capacity as a “healthy setting for living, learning and working” [80]. In the studies we identified, attempts at achieving similar aims involved either integrating health and social care practitioners into the school workforce [57, 58], or by using additional funds to finance a number of health promotion interventions around diet and exercise [55, 56]. Both approaches reported positive effects on health and well-being but with negative consequences on teacher workload [55–58] reflective of the findings of other types of school-based health interventions [81, 82]. In these cases they reported promising improvements in anxiety, mental health, asthma management and vision screening [81] but with the impact on educational outcomes and constraints of staff resource hindering sustainability [82]. There is evidence that if these school-based initiatives are to be successfully sustained their implementation must accommodate intrinsic factors relating to school-specific autonomy, dedicated staff engagement initiatives and community support as well as contextual conditions relating to time, funding and external project support [83]. There is also a more fundamental issue that such school-based interventions fail to address, which is that their attempts at reaching underserved populations is predicated on their regular attendance at school. However this is regularly below national averages for similar socio-cultural reasons that inhibit their engagement with health and social care [84, 85].

### Strengths and limitations

This systematic review was prospectively registered and the identification of studies conducted with reference to best practice by two researchers working independently [31, 86]. The use of qualitative or mixed-method studies alongside quantitative research allows for additional context regards the knowledge, attitudes, and behaviours of clinicians or patients delivering or accessing the service [87]. Despite the comprehensive search strategy identifying 16 papers the overall quality of the evidence was poor as observed in previous reviews of similar services [25, 26]. Only four of the studies were RCTs and there was little data on outcomes and impact over the medium and long term [45, 46, 48, 49]. The findings were further limited by high drop-out rates [45–47, 49, 50] and a lack of homogeneity, even within model types, precluded a formal meta-analysis and any meaningful comparison of the effectiveness between the three models.

### Implications for policy and research

In light of growing child poverty rates in high income environments [88, 89], the lack of sustained engagement and high drop-out rates reported by many of our studies [45–47, 49, 50] highlights the importance of delivering services co-designed with intended users [46, 49, 90]. The three typologies of integrated service we identified are not intended to be a definitive list and others may emerge including hybrid service offerings that combine elements of each. However, the importance of effective system navigation was understood across all models [45, 47–50, 53], and its importance in accessing and engagement with care is widely recognised both in the UK and elsewhere [9, 91–93].

The establishment of a more robust evidence base is inhibited by the current focus on short-term pilots and funding cycles despite complex interventions needing time to become embedded and medium term outputs that extend beyond the limitations of annual funding cycles that impact on organisations such as the NHS [17, 94]. In response there have been calls for the funding of teams delivering novel services as opposed to funding on project-by-project basis, similarly that health care organisations should redesign structures and processes to promote long-term thinking, incremental delivery and ongoing improvements [95, 96]. Their evaluation also needs to incorporate more precise description of the service model, and the measurement of outcomes valued to both service and patient using mixed methodologies and some element of cost effectiveness [97, 98].

## Conclusion

There are many challenges to integrating the delivery of health care and social support for children and there is much to learn. While this review has confirmed a lack of robust evidence for the benefits of integration for CYP from underserved populations there are promising indications of a number of positive impacts and the nascent typology of services offers some structure to further understand the differences and similarities between models. To fully understand their potential, more robust evaluation methods are needed of services that are commissioned for longer periods of time and which are able to be flexible and culturally adaptive in their attempts to engage with underserved communities.

## Electronic supplementary material

Below is the link to the electronic supplementary material.

Supplementary Material 1

## Data Availability

This is a review of previously published data.
